# Neurophysiology of male sexual arousal—Behavioral perspective

**DOI:** 10.3389/fnbeh.2023.1330460

**Published:** 2024-01-24

**Authors:** Wiktor Bogacki-Rychlik, Katarzyna Gawęda, Michal Bialy

**Affiliations:** Department of Experimental and Clinical Physiology, Laboratory of Centre for Preclinical Research, Medical University of Warsaw, Warsaw, Poland

**Keywords:** sexual arousal, sexual behavior, sexual motivation, sexual preference, male

## Abstract

In the presented review, we analyzed the physiology of male sexual arousal and its relation to the motivational aspects of this behavior. We highlighted the distinction between these processes based on observable physiological and behavioral parameters. Thus, we proposed the experimentally applicable differentiation between sexual arousal (SA) and sexual motivation (SM). We propose to define sexual arousal as an overall autonomic nervous system response leading to penile erection, triggered selectively by specific sexual cues. These autonomic processes include both spinal and supraspinal neuronal networks, activated by sensory pathways including information from sexual partner and sexual context, as well as external and internal genital organs. To avoid misinterpretation of experimental data, we also propose to precise the term “sexual motivation” as all actions performed by the individual that increase the probability of sexual interactions or increase the probability of exposition to sexual context cues. Neuronal structures such as the amygdala, bed nucleus of stria terminalis, hypothalamus, nucleus raphe, periaqueductal gray, and nucleus paragigantocellularis play crucial roles in controlling the level of arousal and regulating peripheral responses via specific autonomic effectors. On the highest level of CNS, the activity of cortical structures involved in the regulation of the autonomic nervous system, such as the insula and anterior cingulate cortex, can visualize an elevated level of SA in both animal and human brains. From a preclinical perspective, we underlie the usefulness of the non-contact erection test (NCE) procedure in understanding factors influencing sexual arousal, including studies of sexual preference in animal models. Taken together results obtained by different methods, we wanted to focus attention on neurophysiological aspects that are distinctly related to sexual arousal and can be used as an objective parameter, leading to higher translational transparency between basic, preclinical, and clinical studies.

## Introduction

Understanding the mechanisms that control particular types of behavior requires appropriate models. In the case of sexual behavior, the evolutionary conservative character of regulatory loops qualifies animal models as homologically reliable (Pfaus et al., 2003). This phylogenetic continuance is visible in similar across-species hormonal regulation (mainly steroid sex hormones) and neuroanatomical correspondence. To this day, the most studied animal models of sexual behavior are rats (Rattus norvegicus), and thus, in this paper, if there is no additional mention of other species, the presented data refers to rats. Above its translational value, basic and preclinical studies of rat sexual behavior give insight into many aspects of general neurobiology, physiology, and pharmacology by providing reliable and repeatable parameters.

Despite its conservative and highly innate profile, neurons involved in sexual behavior are subject to neuronal plasticity processes (Hull and Dominguez, 2007). These changes are related to perinatal critical periods during brain development (Gorski, 1985; Baum, 2009). In adult animals, plasticity processes are visible during the acquiring of sexual experience (Bialy et al., 1992, 2000; Bialy and Kaczmarek, 1996; Dominguez et al., 2006; Hull, 2011; Sanna et al., 2017, 2020) adjusting sexual performance to environmental conditions. Typically, during the repeated acquisition of experience and condition to the rewarding value of copulation, the sexual interactions become more effective (shorter intromission and ejaculation latencies) (Larsson, 1959; Dewsbury, 1969).

Sexual interaction, especially copulation to ejaculation, activates the reward system. From highly euphoric to merely responsive, the male climax with the ejaculation reflex is one of the most (if not the most) natural-rewarding states. By using conditioned place preference tests (CPP), there is a possibility to depict the rewarding state of particular phases of sexual activity (Tenk et al., 2009). These rewarding properties can also be used to instrumentalize reaction and, by that, measure the motivational aspect more than the level of sexual arousal (SA) (Whalen, 1961; Hård and Larsson, 1968). Inversely, conditioning processes can also inhibit sexual interactions when punishment processes are applied (Beach et al., 1956).

Regulation of mating behavior is strictly related to hormonal activity, with the HPA-axis and gonadal sex steroids as the center of the regulatory mechanism (Awoniyi et al., 1993; Babaei-Balderlou and Khazali, 2016). Sexual behavior has opened the possibility of identifying sexual-related neuronal loops by testing their responsiveness to steroids (see for review: Sachs and Meisel, 1988). As it turned out, neurons containing receptors for sex hormones themselves do not transfer crucial information for activation of copulation (for instance, odor cues) but instead participate in local loops that modulate and amplify such information (Wood and Newman, 1995; Maras and Petrulis, 2010; Petrulis, 2013; Gresham et al., 2016; Nakamura et al., 2022).

Activation of sexual responses requests specific cues called attractants. The reception of sexual signals, transduction to CNS, and consequential decoding and integration of information are maintained by various sensory channels. The main olfactory and vomeronasal pathways (Mier Quesada et al., 2023) are dominants in rats (and other rodents), with accessory information conveyed by visual, acoustic, and touch-related fibers. Such sensory stimulation gives a chance to tag crucial for sexual arousability neuronal nuclei, and stimulate them artificially (for instance: optogenetic methods) (Kunkhyen et al., 2017).

As a result of the activation of the sensory system, the effectoric activation takes place. The autonomic nervous system prepares peripheral tissue to initiate and consequently maintain copulation patterns until ejaculation. In the case of male sexual behavior, the most evident peripheral indicator of sexual arousal state is an occurrence of penile erection.

During sexual activity, emotions are also expressed. As always, subjective and objective parts of emotional responsiveness are challenging during their separation. Previously, it has been described that detailed ultrasonic vocalization analysis could serve as an additional parameter depicting these components (Barfield et al., 1979; Bogacki-Rychlik et al., 2021, 2022).

To express the sexual behavior repertoire, there is a need to activate vast regions of the CNS. Thus, the major condition that enables all other specific reactions is the state of so-called general arousal (GA). From a behavioral perspective, if an animal is awake, responsive, and presents proper locomotor (exploratory) activity, we can judge that the level of GA is adequate (similarly to medical examination).

The knowledge accumulated during many years of investigation of animal sexual behavior may also be useful in preclinical research, as many aspects of physiology and pathophysiology related to age, cardiovascular disease, metabolic disease, depression, and others affect sexual activity (Bialy et al., 2019). The study of rat sexual behavior finally matters for understanding human sexuality and behavior (Bancroft, 2008). Despite the general limitation of preclinical transferability, sexual behavior has some more specific restrictions (Le Moëne and Ågmo, 2019), and thus, direct translation of experimental data is challenging.

Furthermore, this problem is enhanced by the non-coherent terminology used by field experts, which implicates a broader misunderstanding when the application of models is trying.

In this review, we focused on the definition of sexual arousal, methods to investigate this state, and its relation to sexual motivation and GA.

We propose to define male sexual arousal as a set of autonomic nervous system (ANS) reactions triggered by specific sensory inputs, analyzed on different levels of CNS, leading to the occurrence of penile erection and potentially ejaculation. This definition emphasizes the importance of physiological reflexes rather than subjective (introspective) psychological reactions. It also gives the conceptual scaffold to analyze arousal by detecting autonomic parameters and separating them from less specific motivational components.

We hope that this terminological clarification, together with reminders of fundamental experiments, will be helpful during experiments with sexual behavior.

### Male sexual arousal—Behavioral perspective

The first studies of sexual behavior started in the XIX century, focusing on amphibians, mostly frogs (see Aronson and Noble, 1945). The amphibian spinal cord has far-reaching autonomy from the higher levels of the central nervous system and can independently regulate animal activity. One of the elements of male copulation–the clasp response can be clearly visible in a frog, which has a preserved spinal cord only.

Tarchanoff (1887) found that clasp response is initiated by stimulation of skin receptors, and its strength is proportional to the filling level of seminal vesicles (SVs). When a male, with spared only a spinal cord had filled on seminal vesicles, clasp response was strong. Even after the removal of the heart, liver, kidneys, or testis, this reflex remained to be properly expressed and disappeared shortly before death. In more physiological situations, clasp response disappeared only when SVs were empty and could be restored by filling seminal vesicles with milk (Tarchanoff, 1887). These results suggest that internal sensory information from seminal vesicles passed via the autonomic nervous system is essential to maintain copulation, at least at the level of the spinal cord, and can be the element of sexual arousal. However, amphibians copulation differs compared to mammals activity in many aspects. For example, this group of animals has distinct genital anatomy adapted to external insemination. Their central nervous system is composed of relatively few neurons that mostly occupy ventricular and periventricular regions with poorly visible neuronal clusters (nuclei) without developed neocortex. Hence, there are no close homologs with mammalian brain nuclei (Wilczynski, 2009). Also, the amphibian ANS is poorer developed in comparison with the mammalian system (Biaggioni et al., 2022). Nevertheless, even in this group, different aspects of sexual activity are controlled by various regions of the central nervous system, creating a relatively complex system (Aronson and Noble, 1945).

In mammals, a more comprehensive analysis of male sexual activity started from Frank Beach’s research on rat sexual behavior. Beach (1942, 1956) described that initiation of copulation did not correlate with other behavioral parameters describing copulatory performance. He has proposed to distinguish two independent mechanisms: the sexual arousal mechanism (SAM) and the intromissive-ejaculatory mechanism (IEM). SAM initiates sexual interaction and can be measured by mount latency (time from the introduction of receptive female to first attempt to copulation—mounting). Consequently, IEM regulates copulation and can be measured by ejaculation latency (time from first intromission to ejaculation). Future factor analysis of all sexual parameters distinguished five independent groups of behavioral parameters: (1) anticipatory behavior (measured by level changes in the bi-level cage), (2) initiation of copulation, (3) copulatory efficiency (measured by ejaculation latency), (4) a number of intromissions required to achieve ejaculation, and (5) intromission (hit) ratio (number of intromissions to all attempt copulation). Thus, every group reflects the activity of different neuronal regulatory loops (Sachs, 1978; Dewsbury, 1979; Pfaus et al., 1990).

It’s important to notice that these statistically defined independent factors (groups) do not enable us to fully separate SM from SA (Bialy et al., 2019). However, it is now concluded that initiation of copulation measured by mount latency describes sexual motivation rather than sexual arousal (Hull et al., 2002).

Sachs (2000) pointed out another important factor indicating the complexity of male sexual behavior: the diversity of erection mechanisms. This diversity was visible not only by changes in behavioral contexts but also by various reactions to hormonal stimulation required for erection restoration after castration. To be precise:

- The occurrence of erection obligated to restore initiation of copulation (intromission latency; the time between the introduction of female to the first intromission) depends on estrogen (estradiol or testosterone therapy) (Meisel et al., 1984).
- In contrast, touch-based (ex-copula) erection depends on androgens - dihydrotestosterone or testosterone treatment (Meisel et al., 1984).
- Also, erections during non-contact tests after castration depend on androgens (dihydrotestosterone or testosterone) given peripherally (Manzo et al., 1999).

**FIGURE 1:**
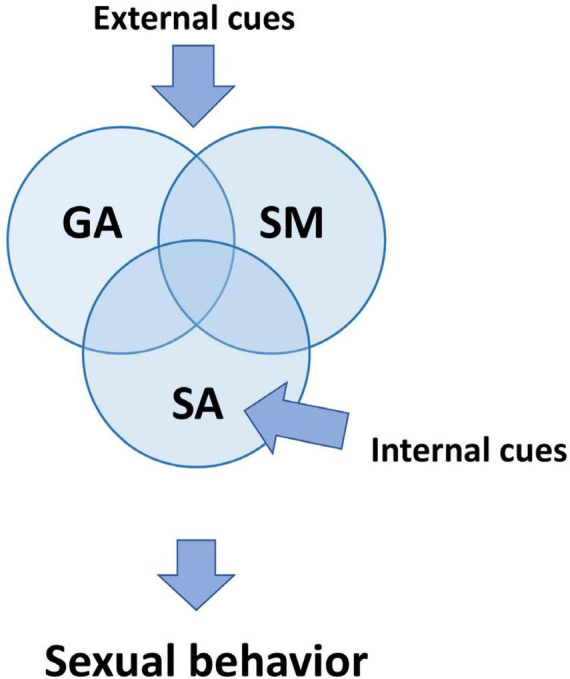
Three primary processes regulating sexual behavior. Although all three processes are defined in the text as separate mechanisms, they are interconnected and affect one another. Sensory cues from genital organs, referred to as internal cues, primarily affect sexual arousal (SA). External cues related to the sexual context have an impact on all SM, SA, and GA. GA is additionally activated by non-specific cues which induce arousal and wakefulness. SA, sexual arousal; GA, general arousal; SM, sexual motivation.

We can see that different observable aspects of male sexual behavior have even more complex inner regulatory composition. This complexity emerges from the parallel and subsequent processing of different neuronal modules. Some of them are specific, and others are not for sexual reactions. During sexual behavior experiments, we can thus observe that particular behavioral components are more associated with some combination (Figure 1) of GA, SA, and SM (Bialy et al., 2019).

Distinctions between sexual arousal and motivation (Sachs, 2000, 2007) and between general arousal and sexual arousal (Bancroft, 1999; Pfaff et al., 2002; Heiman and Pfaff, 2011) were generally accepted during discussions on sexual arousal in the Hormones and Behavior journal [2011, vol. 59(5)].

What do we exactly mean by the mention of male sexual arousal? In the literature, we can come across many terms that refer to sexual arousal. Especially when describing human sexuality, the terminology differs significantly from that used in animal studies. It’s owing to the elaborate description of emotional states proposed by psychologists and sexologists (Janssen, 2011). For instance, the term “sexual desires” may not be entirely proper in animal studies. Sachs (2007) tried to unify terminology that would allow achieving greater translational of animal studies (by comparison with sexological descriptions). He indicates that penile erection is accepted as the golden parameter that measures elevated sexual arousal in males. In the case of psychogenic erections, he proposed that two conditions must be met: (1) the occurrence of penile erection and (2) sexual context. He also proposed (Sachs, 2000, 2007) to distinguish between two processes: sexual arousal and sexual motivation.

Motivation is a process “that causes an organism to seek a goal,” and sexual motivation is “impetus arising from internal or external stimulation to seek out or create occasion for engaging in sexual behavior.” Motivation manifests itself through the rate of executing a response that changes the probability of the occurrence of an unconditioned stimulus (or the signal stimulus of an instinctive behavior) or the conditioned stimulus (in second-order reinforcement). Manifestation of motivation level is conditioning type II also, named as instrumental reaction (Konorski and Miller, 1937; Konorski, 1967) or alternatively “operant behavior” proposed by Skinner (1938). Thus, measurements of the instrumentalized reaction (including frequency, latencies, and persistency) could serve as a reflection of motivation level. In laboratory research of rats’ sexual behavior, different responses can be used, but usually, there are bar-pressing or runway, rewarded by contact to mountings, intromission, or ejaculation (Beck, 1971, 1974, 1997; Everitt et al., 1987, 1989; Beck et al., 2002).

These motivational regulations were profoundly studied using the pharmacological, electrophysiological, and optogenetic methods and are related mainly to the activity of the dopaminergic system of the ventral striatum. Although it seems to be non-specific for sexual behavior, maintaining the intrinsic system of processing all seeking behavior related to conditioned or unconditioned stimuli. Reciprocal connections of the mesolimbic pathway with more sexual-specific structures such as MPOA enable channeling motivational (reward-related) response and initiate sexual activity (Everitt et al., 1989). By using the anticipatory paradigm (Bogacki-Rychlik et al., 2021), mentioned before second-order conditioning or pharmacological modulation (Rodríguez-Manzo and Canseco-Alba, 2023) of the ventral striatum, there is a possibility to partially separate the motivational component from the sexual arousal activation.

**FIGURE 2:**
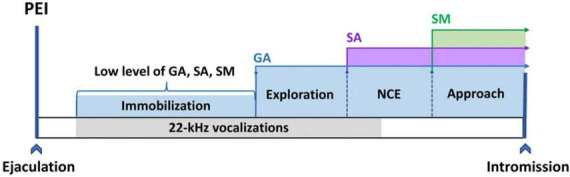
The GA, SA, and SM activation time sequence during the post-ejaculatory interval. Immobilization reflects a low level of GA, SA, and SM. The occurrence of exploration indicates activation of GA.; First NCE (non-contact erection) is associated with an increase in SA; The build-up in SM leads to the manifestation of approach behavior.

In a more natural paradigm, dissociation between general arousal, sexual arousal, and sexual motivation can be visible, for instance, during the post-ejaculatory period (PEI, see Figure 2) based on results obtained from Sachs and Bialy (2000). About half a minute after the first ejaculation (and slightly later after followings), male 22-kHz vocalizations can usually be observed simultaneously with natural immobility (Barfield and Geyer, 1972; Sachs and Barfield, 1976). Such 22-kHz vocalization reflects a relaxation state (Bialy et al., 2016), which occurs after a rapid decrease of arousal (Bell, 1974), at the time when sleep-like spindles in EEG can be recorded (Kurtz and Adler, 1973; Barfield and Geyer, 1975). Taking together all these data, we can assume that about 1 min after ejaculation, general arousal, sexual arousal, and sexual motivation are at a very low level. As the post-ejaculatory period continues, a rise in locomotion activity is observable. This locomotion is manifested as exploratory behavior, focused on the experimental chamber but not on the approach to the female. This movement is mainly related to an enhanced level of general arousal. Concomitantly, the level of sexual motivation and sexual arousal stays low. Only later, the occurrence of non-contact erection indicates an elevated level of SA. However, there is still lack of approaching to the female. In the last phase of PEI, the male starts to approach and investigate the female, which depicts the elevation of SM and initiation of copulation (Sachs and Bialy, 2000; Bialy et al., 2019). The initiation of copulation ends the PEI. Currently, all processes GA, SA, and SM are back on the high level (Figure 2).

Likewise, the dissociation between SA and SM could be observed during the first initiation phase after the anticipatory phase (the time between the introduction of the female to the first mount). At that time, some males also can display non-contact erection. It’s observed more frequently in sexually naïve males, and this type of erection can be visible without subsequent copulation (personal observation). In humans, dissociation between sexual arousal and sexual motivation can be observed in asexual men. They tend to avoid sexual intercourse with partners, although the frequency of masturbation is on the average level of the male population (Portillo and Paredes, 2019). Furthermore, we can distinguish, during behavioral tests, the motivational component by measuring only the approach behavior (Le Moëne and Ågmo, 2019). In this phase, a male presented an oriented reaction toward the sexual-related cues. In other words, his reactions lead to greater sensory stimulation. During approach behavior, the autonomic parameters related to sexual arousal could be absent (including erection). Also, after the approaching phase, the initiation of copulation and/or copulatory phase could not be observable. Describing this phenomenon in the context of classic sexual behavior parameters we can notice: parameters of the instrumentalization of reaction, but improper latency and number of intromission, and consequently often lack of ejaculation (we discuss these changes in Bialy et al., 2019). We can see that if there is a possibility to observe the separation of motivational and SA-related parameters, there are at least two separate interconnected neuronal loops. Physiologically, one of them leads to a greater probability of object-related interaction (motivational aspect), and the other controls the sexual-performance autonomic reactions (sexual arousal aspect).

General arousal and sexual arousal are closely interlinked processes as the reduction of GA (pharmacologically) decreases SA, and elevated sexual arousal increases general arousal (Pfaff, 2017). On the other hand, not every rise in general arousal automatically increases sexual arousal. From a neurophysiological point of view, GA is a result of the activation of an ascending neuronal web consisting of, e.g., reticular formation with upper pons and mesencephalon with noradrenergic, serotonergic, and cholinergic neurons, hypothalamus with histamine and hypocretin (orexin) neurons and thalamus. Disruption in the hypocretin system is a suggested mechanism of narcolepsy (Adamantidis et al., 2020). Lesions in these areas could eventually lead to coma in humans (Silva et al., 2010; Kandel, 2013). The disruption of the activity of the thalamocortical pathways is a proposed mechanism of deep-sedative drugs used in anesthesiology (Malekmohammadi et al., 2019). The activation of GA can also be visualized by the arousing effect of the pain stimulus during the Glasgow Coma Scale examination (Todd et al., 2017). The behavioral reflection of GA is visible by all non-specific reactions, often with a reflexive component, which corresponds with a normal state of awakeness. In rats, we can describe it by, e.g., total locomotor activity during exploration of the cage, number of rearing, and sometimes climbing or digging.

The influence of GA during sexual interactions was pictured in some experiments with pain stimulation. Results showed that moderate painful shock can promote copulation (approach to female and intromission) (Barfield and Sachs, 1968; Sachs and Barfield, 1974). Interestingly, during PEI, moderate pain effectively shortened this phase but only when applied after the end of 22-kHz vocalizations. It was ineffective or even prolonged post-ejaculatory period (PEI) when shocks were applied during 22-kHz emission (Pollak and Sachs, 1975). Therefore, it is possible that GA can stimulate processes that are the most active at that moment. When inhibition during the first part of the post-ejaculatory interval dominates, then general arousal primarily enhances inhibitory processes, but when the elevation of SA starts to be observed, general arousal stimulates sexual arousal and potentially sexual motivation. The same problem can be observed in clinics in patients with an enhanced anxiety level, which has an ambiguous effect on sexual behavior (Janssen and Bancroft, 2007). Anxiety can activate general arousal and, in this way, sexual arousal (Bancroft, 1999), but high anxiety levels can evoke erection dysfunction as well as premature ejaculation (Janssen and Bancroft, 2007). On the other hand, anxiety inhibits sexual motivation and initiation of copulation via neuronal networks in the nucleus accumbens related to initiation of copulation, activated by PKA (protein kinase A), CREB (cAMP response element-binding protein), and D1 receptor (dopamine receptor type 1) (Barrot et al., 2005; Bialy et al., 2010).

### Male sexual arousal—Core assumptions

It remains to ask what sexual arousal means and whether we can distinguish specific neuronal loops relevant to it. We need to make basic assumptions, which are listed below.

1. We should assume that the most evident indicator of sexual arousal in males is penile erection.

If we assume the above implicatively, we have to consider that:

- The basis of sexual arousal is neuronal loops consisting of the afferent sensory inputs at the level of the spinal cord, creating synapses with efferent autonomic fibers responsible for erection and ejaculation.
- These loops are regulated by higher neuronal systems, creating reciprocal interactions at the three levels of the autonomic nervous system.

Thus, the second assumption is:

2. The activity of the sexual-related ANS neuronal structures can serve as a parameter describing male sexual arousal.

### Levels of the autonomic nervous system

#### Mechanism of erection

**FIGURE 3:**
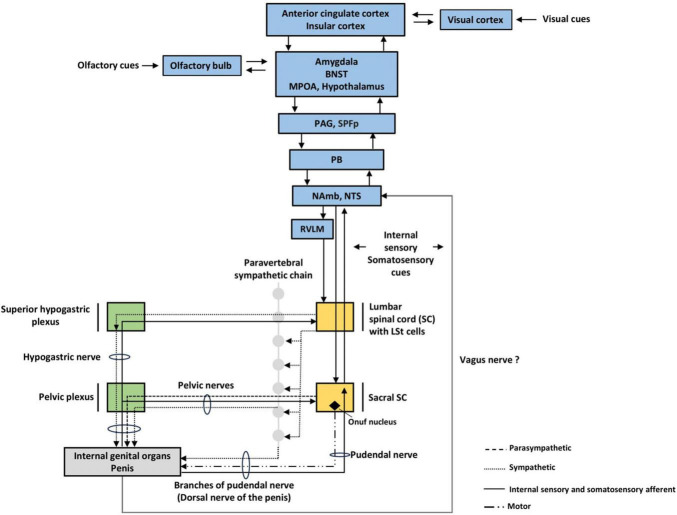
Autonomic nervous system (ANS) structures involved in sexual arousal. We pointed out three levels of ANS. Visual cues are more likely to elevate sexual arousal in humans, while rats rely more on olfactory cues. BNST; bed nucleus of stria terminalis, LSt, lumbar spinothalamic cells, MPOA, medial preoptic area; PAG, periaqueductal gray; SPFp, parvocellular subparafascicular thalamic nucleus; PB, parabrachial nucleus; NAmb, nucleus ambiguus; NTS, nucleus tractus solitarius; RVLM, rostral ventrolateral medulla.

**FIGURE 4:**
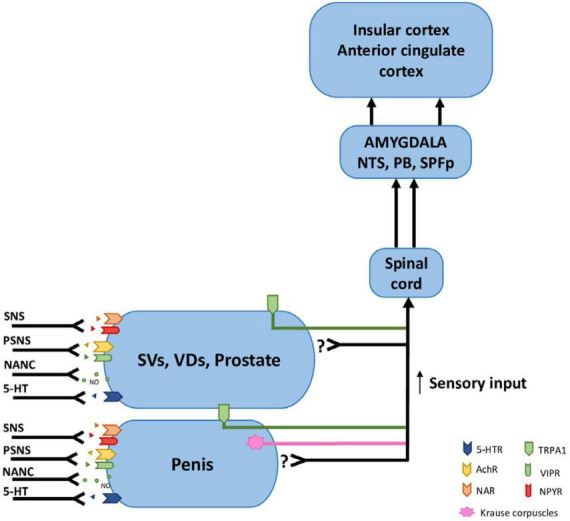
Receptors, afferent and efferent innervation of the internal genital organs and penis. TPRA1 receptors innervate internal genital organs as well as the penis. Krause corpuscles innervate the penis. The main afferent pathways conducting sensory signals from internal genital organs travel along the pudendal nerve, hypogastric nerve, and pelvic nerves. The efferent pathways that innervate the internal genital organs and penis contain sympathetic fibers with NA (noradrenergic) and NPY receptors, parasympathetic with Ach (cholinergic) and VIP receptors, NANC with nitric oxide (NO), serotoninergic with 5-HT receptors.; SVs, seminal vesicles; VDs, vasa deferentia; NTS, nucleus tractus solitarius; PB, parabrachial nucleus; SPFp, parvocellular subparafascicular thalamic nucleus; 5-HTR, Serotonin receptor; AchR, Acetylcholine receptor; NAR, Noradrenergic receptor; TRPA1, Transient receptor potential cation channel subfamily A member 1; VIPR, Vasoactive intestinal peptide receptor; NPYR, Neuropeptide Y (tyrosine) receptor; NANC, Non-adrenergic Non-cholinergic fibers; SNS, Sympathetic Nervous System; PSNS, Parasympathetic Nervous System.

The penile erectile tissue consisting of pair corpora cavernosa and corpus spongiosum have a complex sinusoidal blood system innervated atypically by parasympathetic, sympathetic, and nitrinergic NO fibers (non-adrenergic non-cholinergic fibers) (Giuliano et al., 1995). The rareness of it arises from the fact that most blood vessels are innervated only by sympathetic fibers with dominantly adrenergic receptor expression. Adrenergic stimulation participates in vasoconstrictive response, typically maintaining redistribution of blood flow with opposite local mechanisms of paracrine vasodilation as an antagonistic lever. In opposition to that, sexual performance requires all autonomic types of fibers to be active simultaneously, creating probably the most complex autonomic response (Figures 3, 4). It seems that the foremost neurons responsible for the intensity of erectile performance are pelvic nerves located in the sacral segment of the spinal cord, which contains all types of necessary fibers. The parasympathetic stimulation leads to the secretion of acetylcholine from postganglionic fibers, and thus, by activating the M-type receptors on the vascular epithelium (endothelium) generates the vasodilatory effect by (nitric oxide) NO production. This effect is mediated simultaneously by the secretion of VIP and adenosine. Similarly to parasympathetic stimulation, non-adrenergic non-cholinergic fibers directly secrete NO, thereby contributing to evoke erection in men and rats (Hull et al., 1994; Andersson and Wagner, 1995; Bialy et al., 1996).

At the same time, sympathetic stimulation increases, triggering the cardiovascular system. It leads to positive tropic effects on heart parameters (positive chrono-, ino-, and dromotropic effect), with an increase in heart rate and stroke volume. By adrenergic stimulation of the vascular component, this response constricts most of the body’s blood vessels (including pelvic arteries) and raises total resistance and blood pressure. In penile arterioles, in a rest state, adrenergic activation inhibits the erection in this vasoconstrictive mechanism. However, parasympathetic and nitrergic coactivation, together with local mediators, e.g., prostacyclin, reduce the contraction of the supply arterioles and provide sufficient fill of the penile corps. Activation of the sympathetic nervous system also determines another parameter of sexual arousal, which is ejaculation. It does so by innervating organs participating in ejaculation: the vas deferens, seminal vesicles, prostate, and testicles (Giuliano, 2011; Clement and Giuliano, 2016).

The next important mechanism refers to the atypical regulation of muscle tension in the pelvic floor group. In humans, contraction of these muscles blocks blood outflow by pressing on the vein system. In rats, the quick muscular response provides rather more inflow than inhibits the blood return. Despite some physiological differences between species, the contraction of ischiocavernosus and bulbospongiosus muscles during sexual activity is coordinated by the autonomous pathways on the spinal level (Hull et al., 2002).

Thus, we can see that the most apparent arousal parameters are functions of autonomic neuronal efferent activation (output). So, the question is, which receptors and afferent sensory inputs of these reflexes are necessary to activate the neurons of the sacral segment of the parasympathetic nervous system?

#### Sensory receptors

Surprisingly, such receptors are relatively poorly investigated (Figure 4). Krause corpuscles are one of the receptors detected on the glans penis. They are rapid-adapting low-threshold mechanoreceptors, sensitive to light touch and vibration (optimum 40–80 Hz). Activation of these receptors induced penile erection, and genetic ablation of Krause corpuscles impaired intromission and ejaculation in mice (Qi et al., 2023). In men, the glans penis stimulation induced contraction of the cavernous muscle, the smooth muscle of the seminal vesicle, and the vassal ampullary. These reflexes were inhibited by the glans penis, cavernous muscle, seminal vesicle, and vassal ampulla anesthesia (Shafik et al., 2007). However, very little is known about internal sensory input from seminal vesicles or other parts of internal genitalia and their role in autonomic nervous system activity in mammals. In rats, can be observed erections named touch-based (ex-copula) that can be evoked by constant pressure induced by a lead stick transverse to the penile root. Such pressure can be detected at the internal genital tract as well (seminal vesicles). Ex-copula erections can be induced after a relatively long time–a few minutes (Sachs and Bitran, 1990). It differed from erections generated during copulation by stimulation of the glans penis during intromission with rapid bulbocavernosus muscle contraction and very quick erection (at the time less than 0.5 s).

If the impulse from the internal genitalia can elevate sexual arousal, is it possible for a male to have an elevated level of sexual arousal without an explicit sexual context, but only as the effect of activation of these internal receptors? In the frog, such information is crucial for the clasp reflex (Tarchanoff, 1887), but innervations of internal organs can differ between groups of animals. So-called vacuum activity (“germ. Leerlaufreaktion”) proposed by Konrad Lorenz, can suggest that information from internal organs can induce elevated sexual arousal. This term means that an animal discharges an elevated level of sexual arousal on an inappropriate object and in an inadequate situation. This reaction is sometimes seen in dogs that mount, for example, plush toys. This mentioned hypothesis is an attempt to understand the highly sexually aroused behaviors of males, with problematic and unclear context from external sexual-related cues. In these cases, we reject other explanations, such as forms of dominance, fetishism, or sexual preference, and can assume that such behavior is related to internal sexual cues.

The critical question about the role of sensory information from internal genital organs on sexual arousal in mammals is currently under verification. The first experiments were not promising, as removing seminal vesicles had no effect on copulation (Beach and Wilson, 1963; Larsson and Swedin, 1971). Beach and Wilson (1963) suggest that the effect of internal genital receptors can be better visualized in animals with strictly seasonal sexual activity. More recently, the connection between the filling state of seminal vesicles and copulation level in sexually naïve mice at the start of the experiment has been found (Birkhäuser et al., 2012). fMRI (functional magnetic resonance imaging) brain scans of men also suggested that the CNS activity could be dependent on the filling state of seminal vesicles (Weisstanner et al., 2022). These results must be considered preliminary, with significant limitations related to a lack of additional control groups. There is a necessity to eliminate an alternative, central mechanism of sexual satiety to avoid misinterpretation. Such an effect could be induced by shifting in the profile of neurotransmission, i.e., dopamine (Hull et al., 1999; Canseco-Alba et al., 2022), serotonin (Matsumoto et al., 1997), noradrenaline (Rodríguez-Manzo and Fernández-Guasti, 1994), opioids (Rodríguez-Manzo and Fernández-Guasti, 1995), GABA (Rodríguez-Manzo and Canseco-Alba, 2017), endocannabinoids (Rodríguez-Manzo and Canseco-Alba, 2023), and glutamate (Rodríguez-Manzo, 2015).

This regulatory mechanism of sexual satiety emerged as a complex interconnected web with differentially expressed neurochemical specificity of particular neuronal clusters that participate in motivational, arousal, and sensory aspects. Thus, it could be challenging to definitely separate the mentioned interoceptive activation, especially in the context of reliable biomedical translation.

Surprisingly, there is very little experimental data on sensory innervation of seminal vesicles. Only one article showed that, in humans, SVs contain multimodal sensory receptors type TRPA1 [transient receptor potential cation channel, subfamily A, member (1) expressed on nerve fibers type C and epithelium]. They were found in fibers with colocalization with nNOS (neuronal nitric oxide synthase) and CGRP (calcitonin gene-related peptide) (Rahardjo et al., 2023). TRPA1 receptors are commonly known to be involved in the reception of stretching, nociception, and inflammation sensations (agonized by, e.g., capsaicin and cinnamon). Despite SVs, TPRA1 receptors are present in the prostate, corpus cavernosum, and glans penis (Figure 4). Thus, these receptors probably participate in neuronal loops that cause smooth muscle contraction and the release of neurotransmitters important in the ejaculation reflex, i.e., NE from sympathetic postganglionic fibers (Birowo et al., 2009); and 5-HT acting probably as a paracrine mediator produces in neuroendocrine cells of SV (Aumüller et al., 2012) and also speculatively of intestinal origin (Jiménez-Trejo et al., 2021).

It is hypothesized that some form of dysregulation in the sensory innervation of genitalia could be one of the pathophysiological mechanisms of premature or delayed ejaculation. SVs are innervated by sympathetic and parasympathetic fibers which contain noradrenaline, acetylcholine, serotonin, and neuropeptides VIP (vasoactive intestinal peptide), PHI (peptide histidine isoleucine) NPY (neuropeptide Y), somatostatins, as well as CGPR and substance P (CGPR and substance P seems to be related to sensory afferent). The function of SVs depends also on androgens treatment (Pinho et al., 1997). Serotonin and augmented levels of this neurotransmitter caused by serotonin selective reuptake inhibitors (SSRI) modulate noradrenergic smooth muscle contraction (Birowo et al., 2010) after hypogastric nerve stimulation (Kim and Paick, 2004) via 5-HT1A, 1B, and 2C receptors (Waldinger et al., 1998). Regulation of serotonin levels is a crucial neurochemical mechanism controlling ejaculation, clearly depicted by pharmacological treatment of premature ejaculation using SSRI and 5-HT1A receptor antagonists (Olivier et al., 2023).

Therefore, the next question regards what pathways conduct impulses from internal organs reaching the higher levels of the autonomic nervous.

#### Spinal reflexes

In humans and animals with an intersected spinal cord, erections are still feasible to induce. Moreover, touch-based erections can be evoked significantly easier in mice and rats with an intersected spinal cord when compared to control animals (Meisel and Sachs, 1980; Sachs and Garinello, 1980). It indicates that inputs coming from higher levels of CNS into the erection center in the spinal cord are predominantly inhibitory. However, after copulation to satiety, the inhibition of erection can’t be abolished by the thoracic spinal cord block. It suggests that such copulation directly inhibits the local spinal intrinsic system (Sachs and Bitran, 1990). Together, these results show that in mammals, spinal reflexes alone are sufficient to induce erection. Analogical results were obtained during experiments with the ejaculatory mechanism, depicting the spinal intrinsic system for ejaculation. The autonomy of this system is underlined by the presence of pacemaker neurons, generating the efferent motoneuronal signal, mediating the contraction of muscles associated with the genital tract (see for review: Carro-Juárez and Rodríguez-Manzo, 2008).

Men with transected spinal cords at the level of T10 still can display some preserved genital reflexes (penile erection) after exposition to pornographic movies (see Sachs, 1995). In this case, the speculative pathway of transduction includes sensory fibers of the hypogastric nerve. The same level of spinal transection in women does not eliminate the ability to achieve orgasm visible in fMRI brain activity during genital stimulation (Komisaruk et al., 2004), with a potential alternative transduction pathway through the parasympathetic vagus nerve (received supraspinal nucleus solitary tract, NTS, and nucleus ambiguus).

#### Supraspinal autonomic nervous system loops—Brainstem level and forebrain

Information from internal organs, in this case from sexual organs, reaches the supraspinal level: lower brainstem with the most important structures such as nucleus solitary tract, nucleus ambiguus, parabrachial nucleus, rostral ventrolateral medulla, nucleus raphe pallidus. The brainstem structures are connected with and regulated by the hypothalamus, amygdala, insular cortex, and anterior cingulate cortex. The amygdala provides tagging of the biological significance of environmental stimuli, correspondingly with the detection of internal information and hormonal state of an organism. The insular cortex integrates multiple internal sensations and, by that, participates in the generation of visceroception and even more general interoceptive awareness. The dorsal posterior insula functions as the primary interoceptive cortex by receiving and integrating inputs from visceral, pain, and thermal receptors via the thalamus, parabrachial nucleus, and nucleus of the solitary tract or nucleus tractus solitarius (NTS). Anterior insula integrates interoceptive signals with emotional and cognitive processes, thus participates in conscious body sensation. The insula is a primary viscerosensory as well as visceromotor area that controls sympathetic and parasympathetic outputs. The insula exhibits functional lateralization, with the right insula dominating in sympathetic activation (Craig, 2003). Anterior cingulated cortex, together with the insula, can work as a prediction error signal (Kleckner et al., 2017) from internal organs with interoceptive experience and via parabrachial nucleus and periaqueductal gray (Lamotte et al., 2021) may convey interoceptive error attenuation. This predictive-signal analysis mechanism, suggests that this area serves an important role in the perception of signal input (rather than strictly reception of it) (Barrett and Simmons, 2015). Thus, dysregulation of this mechanism could hypothetically lead to hyper/hypo-stimulation of lower regulatory loops, resulting in clinically visible sexual dysfunction. Furthermore, it is possible that if this mechanism doesn’t work properly it can contribute to cenesthetic hallucinations, symptoms that sometimes occur in patients with schizophrenia (or during non-schizophrenic psychotic episodes).

### External cues related to sexual context

Sachs (2007) distinguishes between arousal-related erections, which require sexual context, and erections occurring without sexual context (e.g., noctuary erections). He assumed that sexual arousal is not only the activation of the autonomic nervous system–parasympathetic with sympathetic nervous system but also neuronal networks that detect sexually important cues to elevate sexual arousal. This point of view is complementary to that presented herein. However, in most experimental schedules, during exposition to sexual stimuli, similar structures that detect and integrate stimuli control partly both motivational processing and arousing response. Focusing only on autonomic activation allows us to avoid oversimplification and obtain data precisely related to SA.

In vertebrates, sexual arousal consists of processes observable in fish, amphibians, and generally in groups of animals without developed neocortex. These groups of animals have functional evolutionary older areas of the brain like the paleocortex, which constitutes the olfactory system with the olfactory bulb, amygdala, and piriform cortex, and also the archicortex, which forms the hippocampus. These structures play an essential role in the detection of olfactory cues and regulation of sexual arousal and sexual behavior, as well as hormonal status [migration and activity of GnRH (LH-RH) neurons important in gonadotrophin release] (Schwanzel-Fukuda and Pfaff, 1989). In mammals, crucial for regulating sexual behavior structures are the amygdala, bed nucleus of stria terminalis, and medial preoptic area, which create bilateral reciprocal loops. This system displays anatomical and functional sexual dimorphism with high expression of steroid receptors (T, DHT, E, and P) (Hull et al., 2002). Medial amygdala and bed nucleus of stria terminalis provide essential for the elevation of SA processing of sexual-related cues during the occurrence of stimuli (Fiber et al., 1993; Liu et al., 1997; Kondo and Sachs, 2002).

Another source of sensory cues related to the elevation of sexual arousal is tactile cues. The most important ones come from the genital region, which provokes ex-copula erection and erection during copulation. Other tactile cues are also important, especially in sexually naïve rats. For instance, the reduction of tactile cues from vibrissae in sexually naïve male rats significantly reduced the number of males who started to copulate with receptive females (Biały and Beck, 1993). Other cues, like visual and auditory, are also important and can be received on the level of the midbrain as well as the amygdala and neocortex in mammals (Lenschow et al., 2022).

### Neuroimaging study in men and male rats during elevated sexual arousal

Neuroimaging studies of brain activity are mainly based on the measurement of changes in blood flow and neuronal metabolism in various neuronal structures, using PET and fMRI techniques. In rats with stress-induced depressive behavior, erectile dysfunction correlates with alteration activity of such structures as the amygdala, thalamus, hypothalamus, caudate putamen, cingulate cortex, insula, visual-sensory and motor cortices, and cerebellum (Chen et al., 2018). Meta-analysis of men’s brain activity during elevated levels of SA pointed out a high activity of regions related to strictly autonomic sexual-related response, but also other regions corresponding to cognitive, emotional, and motivational components (Kühn and Gallinat, 2011; Stoléru et al., 2012; Poeppl et al., 2014). The data was mainly obtained from tests in which men were exposed to erotic visual stimuli, mainly photos or videos. The right insula (>60% of analyzed articles) and the left anterior cingulate cortex (>60% of analyzed articles) are the structures that increase their activity during elevated SA. The insula is a structure involved in visceral sensory processing (Craig, 2003), and the right insula receives sensory information from the penis and is activated upon genital stimulation (Holstege and Huynh, 2011). The level of insula activation correlates with penile erection rigidity (Moulier et al., 2006; Wang et al., 2017). Additionally, to the insula, the activity of the anterior cingulate cortex (particularly Brock areas 24 and 32) correlates with penile erection, and stimulation of this area can trigger an erection (see Holstege and Huynh, 2011). On the other hand, elevated thalamic activity is observed in >50% of articles, and it may reflect the elevated level of general arousal. Very often, elevated levels of activity have been observed in structures which activity is linked to the analysis of visual stimuli, such as the occipital, parietal, and temporal cortex (Stoléru et al., 2012). It’s worth mentioning that there were no differences in the activity of cortical regions between heterosexual and homosexual men.

Some differences between men and women, as well as heterosexual and homosexual men, can be seen in the hypothalamus response to olfactory stimuli. In homosexual men and heterosexual women, the ventromedial nucleus of the hypothalamus and the preoptic area respond to olfactory cues after exposure to an androgen-like compound. In turn, similar activity of the dorsomedial and paraventricular nuclei of the hypothalamus was detected in heterosexual and homosexual men after exposure to estrogen-like compounds (Savic et al., 2001, 2005).

Ejaculation leads to strong activation of the right insula, dorsal temporal lobe, and globus pallidus. In addition, some structures, such as the amygdala and the parietal, prefrontal, medial orbitofrontal, and temporal cortices, mainly on the left side, can decrease their activity (Holstege and Huynh, 2011).

### Animal model for research psychogenic sexual arousal in males (NCE-test)

**FIGURE 5:**
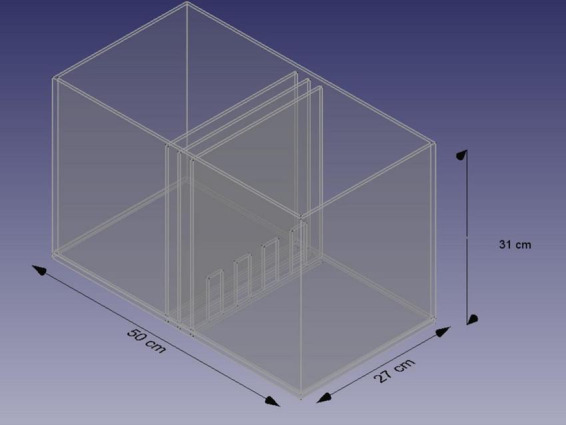
Scheme of the chamber for the NCE test. Based on Sachs et al. (1994) and Bogacki-Rychlik et al. (2022).

Sachs et al. (1994) proposed a new model–a non-contact erections test (NCEs) to investigate sexual arousal in male rats. This type of erection is proposed to be closely related to psychogenic erections in men (Sachs, 1995). In this model, sexual arousal can be measured by the latency to the first erection and the number of non-contact erections during the particular trial, usually 20–30 min. In the NCEs test, the sexual context is well-established and relatively easy to modify, which enables an investigation of sexually arousing factors. Animals in the chamber (Figure 5) are separated, without the possibility of receiving tactile stimulation, but the male is exposed in a controlled manner to the visual, auditory, and odor cues, depending on the barrier type and direction of airflow. This chamber can be modified by various actions. A transparent barrier enables the exposition of visual cues from females. If we would like to reduce the significance of visual cues, there is a possibility to use black walls instead of transparent ones (Sachs, 1997).

To reduce the influence of auditory ultrasonic vocalizations, an additional barrier above the chamber eliminates the detection of vocalizations emitted by another animal (Bogacki-Rychlik et al., 2022).

If we would like to reduce odor cues from the female, we can use sucking air from the female’s side (Sachs, 1997).

Detection of a non-contact erection during trial required direct observation of penile erection. However, if it is not possible, two additional co-occurring elements of male behavior could be used as indicators of such response, i.e., tiptoe with genital grooming (similar to movement and posture observed after intromission) or genital grooming with hip movement. Similar non-contact erections can be observed during the post-ejaculatory period, usually shortly before the end of 22-kHz vocalizations (Sachs and Bialy, 2000; Bialy and Sachs, 2002). Such post-ejaculatory erections can be a more convenient method to analyze NCEs, but with less possibility to modify the sensory environment. On the other hand, during PEI, such NCEs are not influenced by any frustrating context related to the separation of individuals.

Non-contact erections mostly depend on odor cues from a receptive female (Sachs, 1997), detected by the main olfactory system (Kondo et al., 1999). Lesion of olfactory bulbs significantly reduces NCEs (Edwards and Davis, 1997). The medial amygdala seems to be one of the key structures in the regulation of NCEs. Lesion of the medial amygdala eliminates NCEs (Kondo et al., 1997; Kondo and Sachs, 2002). Moreover, the number of non-contact erections correlates with medial amygdala volume (Cooke et al., 2000). A significant effect also can be observed after the bed nucleus of the stria terminalis (BNST) lesion (Liu et al., 1997). Interestingly, a lesion of the medial preoptic area (MPOA) can have no significant effect on NCEs (Liu et al., 1997). It suggests that the main output from the amygdala and BNST important to evoke NCEs is not directed to MPOA. However, MPOA can be involved in NCEs as elevated levels of dopamine in MPOA can enhance NCEs (Adachi et al., 2003), and this structure also regulates the tonus of the sympathetic and parasympathetic nervous system (Hull and Dominguez, 2007).

Paraventricular nucleus (PVN) is a structure that has a direct oxytocinergic output on sacral spinal cord interneurons and can induce penile erection, which can be spontaneous without visible sexual context as well as NCE in a sexual context (Argiolas and Melis, 1995; Melis et al., 1999; Succu et al., 2007). Another structure involved in NCEs is the nucleus accumbens (NAcc). A lesion in the NAcc reduces NCEs (Kippin et al., 2004), and an elevated level of dopamine in NAcc increases the incidence of NCEs (Sanna et al., 2009, 2015). On the other hand, LHA (lateral hypothalamus) exhibits inhibitory effects on NCEs. Lesion located at the anterior part of LHA facilitates NCEs. The same lesion inhibits ejaculation during copulation (Kippin et al., 2004). Lesion of the nucleus paragigantocellularis located in the medulla has no effect on NCEs, although it has a significant influence on reflexive erection and ejaculation (Liu and Sachs, 1999).

Analysis of neuronal activity measured by c-Fos immunostaining showed differences related to NCEs at the NAcc core and shell, medial amygdala (anterior and posterior part), BNST, MPOA (Kelliher et al., 1999), as well as at the orbitofrontal cortex, piriform cortex, PVN, anterior hypothalamus (AHA), ventromedial hypothalamus (VMH), basolateral amygdala (BLA), and central nucleus of amygdala (CeA) (Coria-Avila et al., 2018; Ramírez-Rodríguez et al., 2021). We should keep in mind that elevated C-Fos immunostaining as well as *c-fos* expression do not simply reflect neuronal activity but activity with induction of gene expression and neuronal plasticity (Bialy et al., 1992; Kaczmarek, 1992, 1993, 2018; Bialy and Kaczmarek, 1996) so all active structures can be not visible using this technique.

As we mentioned, NCEs depend on androgens–testosterone (T) and dihydrotestosterone (DHT), but not estrogens (E2) therapy after castration (Manzo et al., 1999; Cooke et al., 2000). Androgens (T and DHT) but not estradiol implanted into sexually dimorphic postero-dorsal medial amygdala maintain NCEs after castration, and latency to first NCE positively correlates with the proximity of androgen implantation to the postero-dorsal region of medial amygdala (Bialy and Sachs, 2002). On the other hand, the blockade of the androgen receptors but not the inhibition of aromatase of testosterone in the postero-dorsal medial amygdala inhibits NCEs (Bialy et al., 2011). The hormonal profile and neuronal network of NCEs differ from the erection observed during copulation. Erection during initiation of copulation (measured by intromission latency) depends on estradiol but not androgen therapy (Meisel et al., 1984; Cooke et al., 2003). Furthermore, estradiol and the activity of aromatase (which transforms testosterone to estradiol) in MeA and medial preoptic area are crucial for erection during copulation (Dominguez et al., 2001; Putnam et al., 2003; Hull and Dominguez, 2007). Results from NCEs tests and copulation show that different networks are involved in erection depending on the context.

Non-contact erection test tests are useful to investigate arousal in the context of sex preference: same-sex, opposite-sex, or juvenile-object (pedophilic-like) preference. In some circumstances (e.g., prenatal inhibition of aromatase of testosterone by letrozole), enhanced same-sex preference can be visible in non-contact tests according to the fact that males display significantly more erections when exposed to other male compared to receptive female. Interestingly, such same-sex preferences in NCEs test do not reflect preference in copulation, in which they prefer to mate with a receptive female (Olvera-Hernández et al., 2015).

In adult males, same-sex preference, or preference to young males, can be evoked by conditioning to male marked by a neutral odor (almond as a CS) associated with D2 dopamine receptor activation. Such conditioning evoked NCEs preferentially when male was exposed to almond-scented male (Coria-Avila et al., 2018) or young juvenile male compared to receptive female. Also, in these experiments, preferences were visible in the NCEs test, but not in the copulation test with receptive female (Ramírez-Rodríguez et al., 2021).

## Summary and implication for future research

In this review, we clarified the term male sexual arousal and its relation to general arousal and sexual motivation. We did this to reconceptualize some issues that emerged from behavioral and physiological results obtained on different models and conceptual paradigms. In this review, we made two core assumptions: (1) penile erection is the most important, pivotal for other parameters, indicator of SA; (2) the activity of parts of ANS that regulate the erection on the spinal and supraspinal level determines the level of sexual arousability. Based on these assumptions, we analyzed autonomic reflexes and a network of interconnectedness of ANS on different neuronal levels. We also visualized these relations by presenting data from brain imaging of humans and rats. Next, we described one of the animal models–the NCEs test, to investigate SA, closely related to psychogenic erections evoked by sexual-related cues. This model is based on top-down regulation of the autonomic nervous system related to SA.

For the sake of clarity and to avoid the informational excessive, we didn’t discuss the mechanism of ejaculation and its relationship to the measurement of SA. Ejaculation has to be considered as the most prominent indicator of sexual arousal accumulation. However, neuronal loops regulating this mechanism especially specific to ejaculation lumbar spinothalamic (LSt) cells (Truitt and Coolen, 2002) are different from erection on discussed levels of a neuronal system (Coolen et al., 2004; Veening et al., 2014; Veening and Coolen, 2014). It’s visible by the fact that erection can be maintained without the triggering of ejaculation, and oppositely, ejaculation can be induced without the occurrence of erection (Clement and Giuliano, 2016). This problem should be discussed more in consecutive review.

From a clinical perspective of urological practice, important questions concerned with the anatomy of innervation of genitals and the physiological construction of reflex arches. To maximize the therapeutic effect while minimizing the side effects, the optimal assessment of the resection range is crucial. Therefore, the knowledge of afferent sensory input and efferent output could answer a question of, e.g., point of resection of seminal vesicles or type of prostatectomy. Paradoxically, innervations of internal genital organs and reflexes related to sexual arousal on the level of spinal cord are relatively poorly understood and require future investigation to complete a picture of down-top and top-down regulation of sexual arousal.

## Author contributions

WB-R: Writing – original draft, Writing – review and editing. KG: Writing – review and editing. MB: Writing – original draft, Writing – review and editing.
